# Genetic Diversity and Virulence Determinants of *Escherichia coli* Strains Isolated from Patients with Crohn's Disease in Spain and Chile

**DOI:** 10.3389/fmicb.2017.00639

**Published:** 2017-05-24

**Authors:** Sandra Céspedes, Waleska Saitz, Felipe Del Canto, Marjorie De la Fuente, Rodrigo Quera, Marcela Hermoso, Rául Muñoz, Daniel Ginard, Sam Khorrami, Jorge Girón, Rodrigo Assar, Ramón Rosselló-Mora, Roberto M. Vidal

**Affiliations:** ^1^Instituto de Ciencias Biomédicas, Facultad de Medicina, Universidad de ChileSantiago, Chile; ^2^Gastroenterology Unit, Clínica Las CondesSantiago, Chile; ^3^Institut Mediterrani d'Estudis Avançats (CSIC-UIB)Illes Balears, Spain; ^4^Department of Gastroenterology and Palma Health Research Institute, Hospital Universitario Son EspasesPalma de Mallorca, Spain; ^5^Department of Pediatrics, University of Virginia School of MedicineCharlottesville, VA, USA

**Keywords:** adherent invasive *Escherichia coli* (AIEC), clonal relationship, Crohn's disease, biopsy, virulence genes, *fimH* mutations, SPATEs

## Abstract

Adherent-invasive *Escherichia coli* (AIEC) strains are genetically variable and virulence factors for AIEC are non-specific. FimH is the most studied pathogenicity-related protein, and there have been few studies on other proteins, such as Serine Protease Autotransporters of *Enterobacteriacea* (SPATEs). The goal of this study is to characterize *E. coli* strains isolated from patients with Crohn's disease (CD) in Chile and Spain, and identify genetic differences between strains associated with virulence markers and clonality. We characterized virulence factors and genetic variability by pulse field electrophoresis (PFGE) in 50 *E. coli* strains isolated from Chilean and Spanish patients with CD, and also determined which of these strains presented an AIEC phenotype. Twenty-six *E. coli* strains from control patients were also included. PFGE patterns were heterogeneous and we also observed a highly diverse profile of virulence genes among all *E. coli* strains obtained from patients with CD, including those strains defined as AIEC. Two iron transporter genes *chuA*, and *irp2*, were detected in various combinations in 68–84% of CD strains. We found that the most significant individual *E. coli* genetic marker associated with CD *E. coli* strains was *chuA*. In addition, patho-adaptative *fimH* mutations were absent in some of the highly adherent and invasive strains. The *fimH* adhesin, the iron transporter *irp2*, and Class-2 SPATEs did not show a significant association with CD strains. The V27A *fimH* mutation was detected in the most CD strains. This study highlights the genetic variability of *E. coli* CD strains from two distinct geographic origins, most of them affiliated with the B2 or D *E. coli* phylogroups and also reveals that nearly 40% of Chilean and Spanish CD patients are colonized with *E.coli* with a characteristic AIEC phenotype.

## Introduction

Crohn's disease (CD) is characterized by chronic inflammation of different sections of the gastrointestinal tract. The etiology of CD is unknown but it has been hypothesized that the interplay of diverse factors, including the intestinal microbiota, genetic, and immunologic host factors, as well as environmental cues, are responsible for its etiology (Kaser et al., 2010). *Escherichia coli* has been shown to occur more often in the gastrointestinal mucosa of patients with CD as compared to non-CD individuals, leading the hypothesis that this microorganism may play a major role in the development of CD (Darfeuille-Michaud et al., 1998, 2004; Schultsz et al., 1999; Ryan et al., 2004; De la Fuente et al., 2014). Furthermore, many of the strains isolated from patients with CD display properties consistent with those exhibited by strains of the adherent-invasive *E. coli* pathotype (AIEC). AIEC strains are able to adhere to and invade epithelial cells, to colonize the intestinal epithelium, and to survive intracellularly in macrophages (Darfeuille-Michaud et al., 1998; Boudeau et al., 1999; Glasser et al., 2001; De la Fuente et al., 2014). The virulence factors harbored by AIEC strains differ from those characteristic of other groups of diarrheagenic *E. coli* strains (Darfeuille-Michaud et al., 1998; Boudeau et al., 1999; Glasser et al., 2001; De la Fuente et al., 2014).

One of the most studied virulence factors in AIEC strains is *fimH*. This gene encodes an adhesin located at the tip of the Type 1 pili; *FimH* mediates bacterial adhesion to glycosylated and non-glycosylated host receptors, including the matrix-associated type I and IV collagens, laminin, fibronectin, and glycosylated receptors (Sokurenko et al., 1997). AIEC that adhere to ileal enterocytes via FimH recognize the carcinoembryonic antigen-related cell adhesion molecule 6 (CEACAM-6) receptor, which appears to be overexpressed in the ileal epithelial cells of patients with CD (Barnich et al., 2007). In addition, polymorphisms in *fimH* confer a higher ability to adhere to CEACAM-6 (Dreux et al., 2013).

Vat-AIEC (Vacuolating autotransporter toxin), a Serine Protease Autotransporters of *Enterobacteriacea* (SPATE) that promotes crossing of the intestinal mucus layer by the LF82 AIEC strain, was recently identified (Gibold et al., 2016). SPATEs constitute a superfamily of trypsin-like serine proteases; they are highly prevalent and are generally secreted by enteropathogens (Yen et al., 2008; Ruiz-Perez and Nataro, 2014). SPATEs have been classified into two groups, class-1 and class-2, based on the amino acidic sequence of their passenger domain and the different functions they perform (Dutta et al., 2002; Ruiz-Perez and Nataro, 2014). To date, class-1 SPATEs have been reported to display cytotoxic effects on cultured cells and enterotoxin activity on intestinal tissues (Boisen et al., 2009; Ruiz-Perez and Nataro, 2014). Most class-2 SPATEs studied display mucinase activity and cleave a variety of leukocyte surface glycoproteins (Ruiz-Perez and Nataro, 2014). The prevalence and possible role of both class-1 and class-2 SPATEs in AIEC strains has received little attention in the literature.

The aims of the present study were to evaluate the genetic variability of 50 *E. coli* isolates from intestinal biopsies of patients with CD in Chile and Spain and 26 isolates from non-CD subjects, to determine the presence of virulence factors typically found in AIEC strains and in strains associated with CD, and to study *E. coli* strains' affiliation with the different phylogenetic groups (A, B1, B2, and D). We also compared the presence of class-1 and class-2 SPATEs and polymorphisms in *fimH* gene, between CD and non-CD *E. coli* isolates.

## Methods

We analyzed 50 *E. coli* strains obtained from patients diagnosed with CD and 26 strains from non-CD patients of which 15 were obtained from subjects who were submitted to colonoscopy for either constipation, anal bleeding, or colon cancer evaluations and had normal colonoscopy results, and 11 were obtained from the feces of normal individuals (Table 1). Forty nine strains (35 from CD and 14 from non-CD patients) were isolated from biopsies taken from 16 patients who underwent colonoscopy at the Son Espases Reference Hospital (Mallorca, Spain) between August 2011 and March 2012; the microbiome of these patients was previously described using molecular methods (Vidal et al., 2015) (Table 1). The Chilean strains were represented by 27 isolates (15 from CD, 1 from a non-CD patient) obtained from patients who underwent colonoscopy at Clínica Las Condes in Santiago, Chile between July 2010 and January 2011, and 11 strains isolated from feces samples of healthy patients (Table 1). All strains isolated from patient biopsies were obtained as previously described (De la Fuente et al., 2014).

**Table 1:** **Description of the two patients groups, Crohn's Disease and Controls, and the number and type of ***Escherichia coli*** isolates**.

**Patient ID^a^**	***E. coli* strains isolate**	**Birth year**	**Origin^b^**	**Year of CD Diagnosis**	**Location^c^**	**Behavior^d^**	**Surgery**	**Medication (at least 3 months before sampling)**	**Clinical status^e^**	**Colonoscopy**
**CD PATIENTS**										
1C	1I06	1975	S	2002	L3	B3	Ileocolic resection	Immunosuppressive agent	Clinical activity	Anastomotic stenosis with ulcers
2C	2C05, 2I05	1963	S	1983	L3	B3	Ileal resection	Immunosuppressive agent	Remission	Normal colon and ileum
4C	4C01, 4I03, 4I01	1955	S	1990	L1	B2	Ileocolic resection	5-aminosalicylates	Remission	Normal colon, anastomotic stenosis
5C	5I01, 5C08	1992	S	2005	L3	B2	Ileocolic	Immunosuppressive agent	Clinical activity	Normal colon anastomotic
6C	6I01, 6I02, 6I06, 6I07, 6I09, 6I10, 6C03, 6C04, 6C09, 6C10	1958	S	2011	L2	B2	Ileocolic resection	5-aminosalicylates	Remission	Normal colon and ileum
7C	7C08, 7C09, 7C02, 7C04, 7C06, 7C07	1983	S	2005	L3	B2	NS	TNF antagononist	Remission	Inflammatory activity in colon and stenosis
9C	9C01, 9C02	1976	S	1984	L1	B3	Ileocolic	TNF antagononist	–	Inflammatory activity in ileum
10C	10C01, 10C05, 10I01, 10I03	1949	S	1997	L1	B1	Ileocolic	5-aminosalicylates	Clinical activity	Normal colon Anastomotic
18C	18I08, 18I02, 18C01, 18C02	1940	S	1940	L1	B3	Ileocolic	Immunosuppressive agent	Clinical activity	Normal colon
24C	24C01	1989	S	2009	L1	B3	Ileocolic resection	Immunosuppressive agent	Remission	Normal colon, anastomotic stenosis
CD43	PT1	1960	CH	1988	L3	B2	NS	NM	Clinical activity	Ileal and colonic ulcers and colon with stenosis
CD45	JSL	1970	CH	2002	L3	B1	NS	Immunosuppressive agent	Remission	Normal colon and ileum
CD44	EII	1985	CH	2008	L2	B1	NS	TNF antagononist	Clinical activity	Normal colon and ileum
CD37	GM	1980	CH	2012	L1	B1	Ileal resection	Immunosuppressive agent	Clinical activity	Ileal ulcers Anastomotic
CD19	FBC	1982	CH	2009	L3	B1	NS	Immunosuppressive agent	Clinical activity	Ileal and rectal ulcers Anastomotic
CD18	CPA	1970	CH	ND	L1	B2	NS	–	Clinical activity	–
CD1	CD1-a	1927	CH	ND	L2	B1	–	NM	Clinical activity	Rectosigmoiditis
CD2	CD2-a	1966	CH	ND	L2	B1	–	NM	No Clinical activity	Normal colon and ileum
CD6	CD6-b, CD6-r	1955	CH	ND	L2	B1	–	Immunosuppressive agent	Clinical activity	Diverticulosis Perianal fistula
CD8	CD8-a	1961	CH	ND	L2	B2	–		Clinical activity	Colon with stenosis Perianal fistula
CD9	CD9-a	1969	CH	ND	L2	B1	–	Anti-inflammatory	Clinical activity	Colitis in the distal segment
CD12	CD12-a	1984	CH	ND	L2	B1	–	–	Clinical activity	Edematous colonic lesions
CD13	CD13-a	1989	CH	ND	L3	B1	–	–	Clinical activity	Colon and ileum active
CD14	CD14-a	1985	CH	ND	L3	B1	–	–	No Clinical activity	Closed fistula
**CONTROL PATIENTS**										
14S	14I01, 14I02, 14C01, 14C05, 14C06, 14C09	1952	S	Control					Constipation	Normal colon
15S	15C02	1965	S	Control					Constipation	Normal colon
16S	16C01, 16C02	1945	S	Control					Hemorrhoid	Normal colon
19S	19C01, 19C02			Control						
22S	22C02, 22C05	1938	S	Control					Hemorrhoid	Normal colon
23S	23C01	1961	S	Control					RCC screening	Normal colon
C7	C7-a	1940	CH	Control						Normal colon
D1-D11	D1, D2, D3, D4, D5, D6, D7, D8, D9, D10, D11		CH	Control					Stool samples	Normal colon

*CD, Crohn's Disease*.

a *Patient ID underlined indicate that they had E. coli strains whose phenotype corresponded to AIEC*.

b *Origin, S-Spain; CH-Chile*.

c *Location, L1-Ileal; L2-Colonic; L3-Ileocolonic*.

d *Behavior, B1-inflammatory; B2-stricture; B3-penetrant disease*.

e *Clinical status, Clinical activity; No Clinical activity; Remission*.

*RCC, rectal colon cancer; NS, no surgery; NM, no medication; ND, no data; TNF, Tumor Necrosis Factor*.

All CD Chilean strains were obtained from ileum biopsies. Almost all Chilean control strains were obtained from stool samples and only one (C7) from an ileum biopsy. The Spanish strains were obtained from colon (C) or ileum (I) biopsies.

Briefly, biopsy samples were suspended in GIBCO® Hank's balanced salt solution (HBSS) (Thermo Fisher Scientific; Grand Island, NY, USA) supplemented with 100 μg/ml gentamicin (Sigma-Aldrich; St. Louis, MO, USA), incubated for 1 h at 37°C and then washed with phosphate buffered saline (PBS) and lysed in 100 μl of 1% Triton-X-100/PBS to release intracellular bacteria. The homogenized tissue obtained was plated on MacConkey agar (Oxoid Ltd.; Basingstoke, Hampshire, UK) and incubated for 18 h at 37°C. Determination and confirmation of *E. coli* identification was first performed using biochemical tests and then by PCR. Study participants provided written informed consent before entering the study. The project and informed consent forms were approved by the Institutional Review Board of Clínica Las Condes; Faculty of Medicine, Universidad de Chile; Ethics Committee of the Northern Metropolitan Health Service, Santiago, Chile; and the Balearic Islands' Ethical Committee, Spain. All records and information was kept confidential and all identifiers were removed prior to analysis.

*E. coli* K-12, NRG857c (Nash et al., 2010), K-12, HS (DuPont et al., 1971; Levine et al., 1978), and HM605 (Clarke et al., 2011) were included as reference strains. All strains were cultured in Luria-Bertani (LB) agar or LB broth (BD Diagnostics; Sparks Glencoe, MD, USA) at 37°C for 18 h.

PFGE was performed according to the protocol described on the PulseNet website of the U.S. Centers for Disease Control & Prevention, using *XbaI* and *SpeI* restriction enzymes (CDC; http://www.cdc.gov/pulsenet/PDF/ecoli-shigella-salmonella-pfge-protocol-508c.pdf). The genetic variability of the CD *E. coli* strains including reference strains HS, reference AIEC strains NRG857c and HM605 (kindly provided by A. Torres and I. Henderson, respectively) and non-CD strains was analyzed by pulse-field gel electrophoresis (PFGE). A *Salmonella enterica* serotype Braenderup (H9812 strain) was used as a gel loading control. PFGE patterns were analyzed with the GEL COMPAR II software (Applied Maths), using the Dice similarity coefficient with a 0.9 % tolerance in band position. Phylogenetic affiliation (A, B1, B2, or D groups) was performed using a previously described method (Clermont et al., 2000). Virulence genotyping was determined by amplifying the following virulence-associated genes commonly present in extraintestinal pathogenic *E. coli* (ExPEC): *cnf1, hlyA, cdtB, neuC, ibeA, papC, sfa/focDE, afa/draBC, fimH*, and *fimAvMT78* (Martinez-Medina et al., 2009), as well as other pathogenicity-associated factors, such as the adhesin synthesis gene (*aufA*), toxins (*ratA, cvaC*), iron uptake (*irp2, fhuD, and chuA*), and Peyer's patch survival (*gipA*). PCR primers used for gene amplification are detailed in Table 2 and PCR amplifications were performed under standard conditions, as previously described (Martinez-Medina et al., 2009).

**Table 2:** **Primers used to PCR analyses for adhesins, iron uptake proteins, toxins, ***E. coli*** phylogenetics groups, SPATEs, and others virulence factors**.

**Primer sequences (5^′^–3^′^)**	**Target sequences**	**References**
**ADHESIN**		
afaBC-F: GCTGGGCAGCAAACTGATAACTCTC	Afimbrial adhesin	Le Bouguenec et al., 1992
afaBC-R: CATCAAGCTGTTTGTTCGTCCGCCG		
aufA-F: TTGGTGGGGCGGATACTGAC	Putative fimbrial-like protein	This study
aufA-R: TGTATAAGCGGCGGTGGAGG		
fimA_v MT78_-F: TCTGGCTGATACTACACC	Variant form of the type 1 major fimbrial subunit	Marc and Dho-Moulin, 1996
fimA_v MT78_-R: ACTTTAGGATGAGTACTG		
fimH-F: GATCTTTCGACGCAAATC	Adhesin of type 1 fimbriae	Arne et al., 2000
fimH-R: CGAGCAGAAACATCGCAG		
papC-F: GACGGCTGTACTGCAGGGTGTGGCG	Pyelonephritis associated pili	Le Bouguenec et al., 1992
papC-R: ATATCCTTTCTGCAGGGATGCAATA		
sfa/focDE-F: CTCCGGAGAACTGGGTGCATCTTAC	S-fimbrial and *F1C fimbriae* dhesion	Le Bouguenec et al., 1992
sfa/focDE-R:CGGAGGAGTAATTACAAACCTGGCA		
**IRON ACQUISITION**		
chuA-F:TATGATGGTCAGCGTTATCGAC	Outer membrane hemin receptor	This study
chuA-R: CATAGCCAGGTTGTTTGCTGTA		
fhuD-F: AACCTTGAACTGCTGACCGAAAT	Component of the ferric hydroxamate uptake (Fhu) system	This study
fhuD-R: GTACTGCCCCAGAAGTTGGTTTC		
irp2-F: CGCAGAATCGCTGTTAGCAC	Iron regulatory protein 2	This study
irp2-R: TGGTAGCGATCTTCAGGGGA		
**TOXINS**		
cdtB-F: GAAAGTAAATGGAATATAAATGTCCG	Cytolethal distending toxin, subunit B	Johnson and Stell, 2000
cdtB-R: AAATCACCAAGAATCATCCAGTTA		
cnfI-F: AAGATGGAGTTTCCTATGCAGGAG	Cytotoxic necrotizing factor 1	Yamamoto et al., 1995
cnfI-R: CATTCAGAGTCCTGCCCTCATTATT		
cvaC-F: CACACACAAACGGGAGCTGTT	Gene codes for the structural gene for colicin V α-Hemolysin	Johnson and Stell, 2000
cvaC-R: CTTCCCGCAGCATAGTTCCAT		
hlyA-F: AACAAGGATAAGCACTGTTCTGGCT	Alfa hemolysin	Yamamoto et al., 1995
hlyA-R: ACCATATAAGCGGTCATTCCCGTCA		
ratA-F: CCGTCATTTTCGCCGCACCT	Ribosome association toxin	This study
ratA-R: ACCCACAGCCAGTCGCAGAT		
**OTHERS VIRULENCE GENES**		
ibeA-F: AGGCAGGTGTGCGCCGCGTAC	Invasion of brain endothelial (IbeA) protein (invasin)	Johnson and Stell, 2000
ibeA-R: TGGTGCTCCGGCAAACCATGC		
gipA-F: TTCCCCTCCAGCAGTCGTTG	Peyer's patch-specific factor	This study
gipA-R: GAACATCCAGCGGCGACTTG		
neuCK1-F: AGGTGAAAAGCCTGGTAGTGTG	K1 capsular polysaccharide	Watt et al., 2003
neuCK1-R: GGTGGTACATTCCGGGATGTC		
pduC-F: GTTGCCGTTGCTCGCTATGC	Coenzyme B12-dependent 1,2-propanediol catabolism	This study
pduC-R: ACTGCACGGATGCCTGATGG		
***E. coli*** **PHYLOGENETIC GROUPS**		
chuA.1: GACGAACCAACGGTCAGGAT	Outer membrane hemin receptor	Clermont et al., 2000
chuA.2: TGCCGCCAGTACCAAAGACA		
yjaA.1: TGAAGTGTCAGGAGACGCTG	Coding for protein of unknown function	Clermont et al., 2000
yjaA.2: ATGGAGAATGCGTTCCTCAAC		
tspE4C2.1: GAGTAATGTCGGGGCATTCA	Tsp encodes for a putative DNAfragment (TSPE4.C2) in *E. coli*	Clermont et al., 2000
tspE4C2.2: CGCGCCAACAAAGTATTACG		
**SPATEs CLASS-1 (Cl-1)**		
Sat1: TCAGAAGCTCAGCGAATCATTG	Secreted autotransporter toxin	Boisen et al., 2009
Sat2: CATTATCACCAGTAAAACGCACC		
sigA 1: CCGACTTCTCACTTTCTCCCG	Exported serine protease SigA	Boisen et al., 2009
sigA 2: CCATCCAGCTGCATAGTGTTTG		
pet 1: GGCACAGAAT AAAGGGGTGTTT	Plasmid-encoded toxin	Boisen et al., 2009
pet 2: CCTCTTGTTTCCACGACATAC		
espP 1: GTCCATGCAGGGACATGCCA	Extracellular serine protease	Boisen et al., 2009
espP 2: TCACATCAGCACCGTTCTCTAT		
espC 1: AGTGCAGTGCAGAAAGCAGTT	Plasmid (pO157)-encoded)	Boisen et al., 2009
espC 2: AGTTTTCCTGTTGCTGTATGCC	EPEC secreted protein C	
**SPATEs CLASS-2(Cl-2)**		
pic 1: ACTGGATCTTAAGGCTCAGGAT	Protease involved in intestinal colonization	Boisen et al., 2009
pic 2: GACTTAATGTCACTGTTCAGCG		
sepA 1: GCAGTGGAAATATGATGCGGC	*Shigella* extracellular protein A	Boisen et al., 2009
sepA 2: TGTTCAGATCGGAGAAGAACG		
tsh 1: CCGTACACAAATACGACGG	Temperature-sensitive hemagglutinin	Boisen et al., 2009
tsh 2: GGATGCCCCTGCAGCGT		
vat 1: AACGGTTGGTGGCAACAATCC	Vacuolating autotransporter toxin	Boisen et al., 2009
vat 2: AGCCCTGTAGAATGGCGAGTA		
eatA1:CAGGAGTGGGAACATTAAGTCA	Autotransporter protein of enterotoxigenic *E. coli*.	Boisen et al., 2009
eatA 2: CGTACGCCTTTGATTTCAGGAT		
EaaA1: GAAGACGAACTGGTTTACGGTG	EaaA from Prophage P-EibA	This study
EaaA 2: GTGGCATTATCAGCATCAATATC		
EcNA114 1: ACTCAGACATGGAAAGGCGGC	EcNA114-C2sp From UPEC NA144	This study
EcNA114 2: CTCCAGTGATGATCCCACCC		

Class-1 (*sat, pet, sigA, espP, espC*) and class-2 (*sepA, eatA, vat, tsh, pic, eaaA, ecNA144*) SPATEs (Dutta et al., 2002; Ruiz-Perez and Nataro, 2014) were PCR detected using previously described primers (Boisen et al., 2009), as well as others described in Table 2.

AIEC strains are characterized by their ability to: (i) adhere to epithelial cells, (ii) invade epithelial cells, and (iii) survive intracellularly in macrophages (Darfeuille-Michaud et al., 1998; Boudeau et al., 1999; Glasser et al., 2001; De la Fuente et al., 2014). Analysis of adhesion and invasion in epithelial cells properties was performed as previously described (Boudeau et al., 1999). Briefly, duplicate 24-well plates containing confluent monolayers of Caco-2 cells were infected at a 10:1 multiplicity of infection (MOI) for 30 min at 37°C in a 5% CO_2_ atmosphere. The cell monolayers were washed three times with PBS, and the adherent bacteria were harvested by lysis with 0.1% Triton X-100, serially diluted and plated on LB agar plates. Results are expressed as the percent of adherent bacteria with respect to the total bacteria present after infection (planktonic bacteria plus adherent bacteria). For the invasion assay, the cell monolayer, previously infected for 30 min, was washed three times and then incubated for 3 h with medium containing 100 μg of amikacin/ml. After 3 h, the cell monolayer was washed three times with PBS and bacteria were harvested by lysis with 0.1% Triton X-100. Bacteria were considered able to survive when the ratio between the number of intracellular bacteria and initial inoculums was ≥ 0.1%.

Analysis of survival and replication of the strains in urine RAW 264.7 macrophages were done according to Glasser et al. (2001) with some modifications. Confluent monolayers of murine RAW 264.7 macrophages were infected at a 10:1 MOI for 2 h and incubated at 37°C in a 5% CO_2_ atmosphere. After 2 h of incubation, the cell monolayer was washed three times with PBS, and then incubated for 1 h with medium containing 100 μg of amikacin/ml. Three and 24 h post-infection, the cell monolayer was washed with PBS and bacteria were harvested by lysis with 0.1% Triton X-100. The number of CFU/ml recovered was determined. Bacteria were considered invasive when the ratio between the number of intracellular bacteria and initial inoculum was ≥0.1%, and considered able to survive when the number of intracellular bacteria recovered at 24 h was comparable to that recovered at 3 h post infection (100%) or similar to those obtained for reference AIEC strains. In addition, *E. coli* strain isolated form CD that was recovered with a similar or higher ratio than NRG857c and HM605, was considered able to replicate intracellularly. All assays were performed in triplicate.

Amplification of *fimH* was conducted with fimH-F and fimH-R primers (Table 2) using High-Fidelity DNA polymerase (Thermo Fisher Scientific; Waltham, MA, USA). PCR products were purified, following the manufacturer's instructions (EZNA gel extraction kit, Omega Bio-Tek; Norcross, GA, USA), cloned into pGEM-T Easy vector (Promega; Madison, WI, USA), and then sequenced by MACROGEN (Rockville, MD, USA). The *fimH* nucleotide sequences were translated to protein sequences and compared to orthologous protein sequences. Phylogenetic reconstruction based on FimH protein sequences was obtained by Kimura 2-parameter method and a phylogenetic tree was plotted using the nearest-neighbor joining method in MEGA software, version 6.0.

We considered adhesins, iron-transporters, and toxins along with other virulence factors as potential biomarker candidates for CD or AIEC strains phenotype. The association between these candidates and Crohn's disease was statistically estimated using hypothesis testing. We decided to test for biomarkers using by two tests: the Chi-squared independence and proportions comparison, and Odds Ratio (OR) computations. Estimations and computations were performed at VassarStats statistical computation website (http://vassarstats.net/index.html).

## Results

### Genomic types among *E. coli* strains

**Figure 1:**
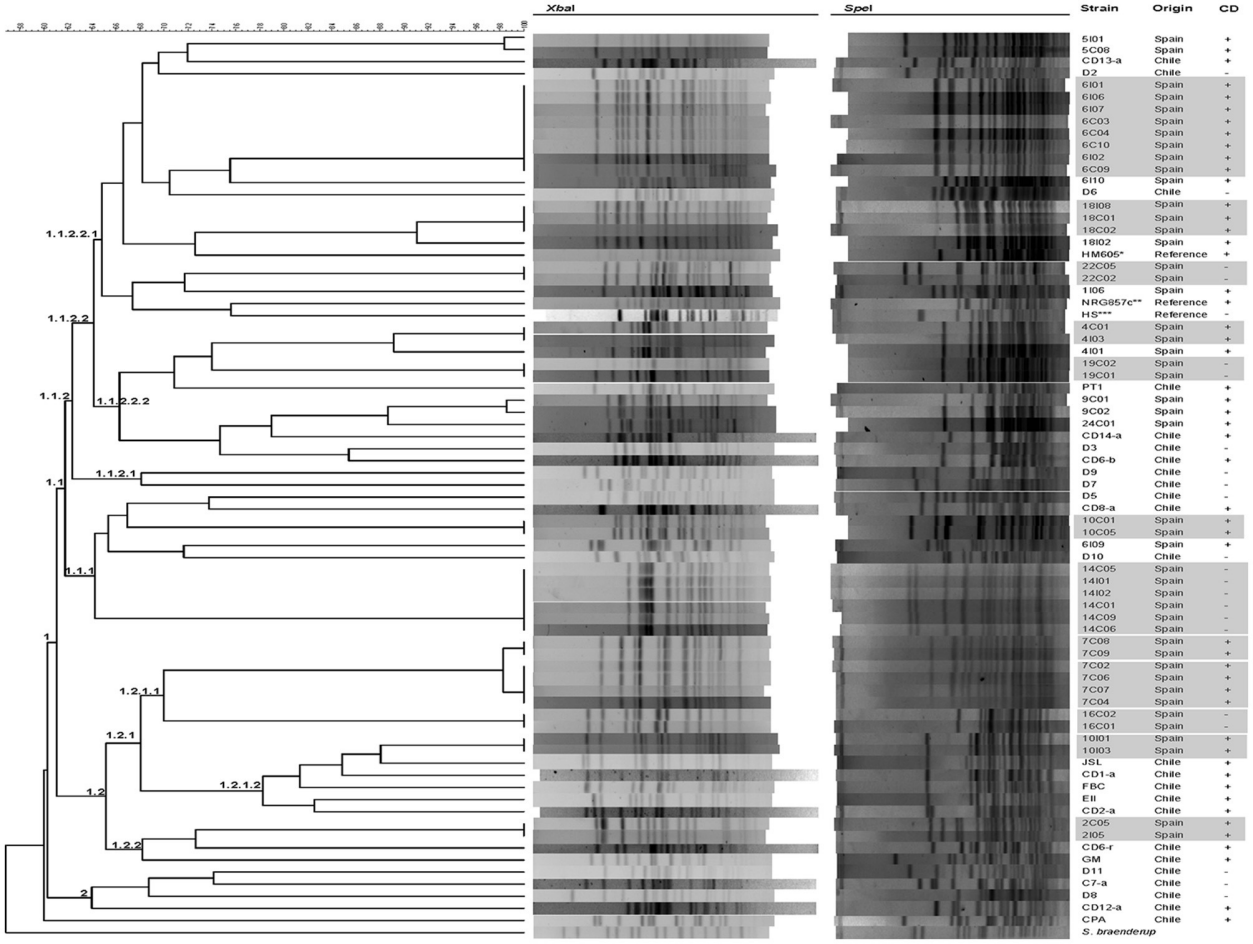
**Clonal relationship between ***Escherichia coli*** strains obtained from intestinal biopsies from patients with CD, biopsies or stool from non-CD patients and reference strains**. The dendrogram based on pulsed-field gel electrophoresis (PFGE) using the *XbaI* and *SpeI* enzymes, allowed the identification of two main groups. Crosses indicate strains isolated from Crohn's disease patients. Clonal related strains are gray shadows highlighted. The reference strains used (Reference) were (^*^) HM605 (AIEC), (^**^) NRG857c (AIEC) and (^***^) HS (commensal *E. coli*).

A set of 76 strains obtained from 24 patients with CD and 18 non-CD patients were included in the present study (Table 1). Seventy one strains were initially selected and analyzed by PFGE using *XbaI* and *SpeI* restriction enzymes. Two main groups (clusters 1 and 2) composed of 70 strains were separated with similarity values >60%. One Chilean strain (CPA) was separated from the other *E. coli* strains studied (Figure 1). The larger cluster (Cluster 1, 66/70) is subdivided into two smaller groups, cluster 1.1, which contains most of the CD and non-CD strains and cluster 1.2, which is composed of 17 CD strains and 2 clonal non-CD strains (16C02 and 16C01). AIEC reference strains NRG857c and HM605 were placed in two branches of cluster 1.1.2.2.1. The smaller cluster (Cluster 2, 4/70), is composed of three non-CD strains (D11, D8 and C7-a) and one CD strain (CD12-a).

In almost all strains, pattern diversity was remarkable, and non-clonal varieties (i.e., identical profiles) were observed, with the exception of PFGE pattern types of strains isolates within a single patient, which were clonal or closely related (i.e., 14C05, 14I01, 14I02, 14C01, 14C06, and 14C09 isolated from patient 14S; 6I01, 6I06, 6I07, 6C03, 6C04, 6C10, 6I02, and 6I09 isolated from patient 6C; 10C01 and 10C05 isolated from patient 10C; 18I08, 18C01 and 18C02 isolated from patient 18C; 4C01, 4I03 isolated from patient 4C; 19C02, 19C01 isolated from patient 19S; 16C02 and 16C01 isolated from patient 16S; 10I01 and 10I03 isolated from patient 10C; 7C08 and 7C09; 7C02,7C06,7C07, 7C04 isolated from patient 7C; 22C05 and 22C02 isolated from patient 22S; 2C05 and 2I05 isolated from patient 2C). From each group of clonal strains, a single strain was selected for further analysis, resulting in 32 non-clonal CD-strains and 18 non-CD (commensal *E. coli*) strains. Of these 32 strains isolated from patients with CD, 13 were of an AIEC phenotype (Table 3). There was no significant difference (*p* > 0.05) in the prevalence of AIECs between Spanish (41.1%, 7/17) and Chilean strains (40%, 6/15).

**Table 3:** **Phylogenetic groups and virulence genes detected by PCR in ***Escherichia coli*** strains isolated from Crohn's disease and control patients (non-CD strains)**.

**Strains**	**phylogroup**	***flmH***	***aufA***	***papC***	***fimAv***	***afaBC***	***Sfa/focDE***	***fhuD***	***chuA***	***irp2***	***ratA***	***cvaC***	***hlyA***	***cnfl***	***cdtB***	***pduC***	***neuCk1***	***gipA***	***ibeA***	**Cl-1**	**Cl-2**	**AlEC phenotype**	**Surgery**	**Immunosupressive agent**	**Medication IFN-γ**	**Clinical activity**	**Remission**
**SPANISH STRAINS**																											
1I06	D	+	–	–	+	–	–	+	+	+	–	–	–	–	–	–	–	–	–	–	–	–	+	+	–	+	–
2C05	B2	+	–	–	–	–	–	+	+	–	–	–	–	–	–	–	+	–	–	–	–	–	+	+	–	–	+
4C01	B2	+	–	–	–	–	–	+	+	+	–	–	–	–	–	+	–	–	–	+	+	+	+	+	–	–	+
4I01	B2	+	–	–	–	–	–	+	+	+	–	–	–	–	–	+	–	–	–	+	+	+	+	+	–	–	+
5C08	B1	+	–	–	–	–	–	+	–	+	–	+	–	–	–	–	+	–	–	–	–	–	+	+	–	+	–
5I01	B2	+	+	–	–	–	–	+	+	+	–	+	–	–	–	+	+	–	–	–	–	+	+	+	–	+	–
6I01	B2	+	–	–	+	–	–	+	+	+	+	–	–	–	–	+	+	–	–	+	–	–	+	+	–	–	+
6I09	D	+	–	–	–	–	–	+	+	+	–	–	–	–	–	+	+	+	–	–	–	+	+	+	–	–	+
6I10	D	+	+	+	–	–	+	+	+	+	+	–	+	+	–	–	+	+	–	+	+	–	+	+	–	–	+
7C02	D	+	–	–	–	–	–	+	+	+	–	+	–	–	–	–	–	–	–	+	+	–	–	–	+	–	+
7C08	D	+	+	–	–	–	–	+	+	+	–	+	+	–	–	+	+	–	–	+	+	–	+	–	+	–	+
9C01	B2	+	–	–	–	–	–	+	+	+	–	–	–	–	–	–	–	–	–	+	+	+	+	–	+	+	–
10C01	D	+	–	–	–	–	–	+	+	+	–	–	–	–	–	+	+	–	–	+	–	–	+	+	–	+	–
10I01	D	+	+	–	–	–	–	+	+	+	+	–	–	–	–	+	+	–	–	–	–	+	+	+	–	+	–
18I02	B2	+	–	–	–	–	–	+	+	+	–	+	–	–	–	+	–	+	+	–	+	–	+	+	–	+	–
18I08	B2	+	+	–	–	–	–	+	+	+	+	–	–	–	–	+	+	+	+	–	+	+	+	+	–	+	–
24C01	B2	+	–	+	–	–	–	+	+	+	–	–	–	–	–	–	–	–	–	+	+	–	+	+	–	–	+
**CHILEAN STRAINS**																											
CD1–a	D	+	–	+	–	–	–	+	+	–	+	–	–	–	–	+	–	–	–	+	–	+	NI	–	–	+	–
CD2–a	D	+	–	+	–	–	–	+	+	–	+	–	–	–	–	+	–	–	–	+	–	+	NI	–	–	–	+
CD6–b	B2	+	–	+	–	+	–	+	+	+	–	–	–	–	–	–	–	–	–	+	+	+	NI	+	–	+	–
CD6–r	D	+	–	–	–	–	–	+	+	–	–	+	–	–	–	–	–	–	–	–	+	+	NI	+	–	+	–
CD8–a	A	+	–	–	–	–	–	+	–	+	–	–	–	–	–	–	–	–	–	–	–	–	NI	NI	NI	+	–
CD9–a	A	+	–	–	–	–	–	+	–	–	–	–	–	–	–	–	–	–	–	+	–	+	NI	+	–	+	–
CD12–a	A	+	–	–	–	–	–	+	–	–	–	+	–	–	–	–	–	–	–	–	–	–	NI	NI	NI	+	–
CD13–a	A	+	–	–	–	–	–	+	–	+	–	+	–	–	–	–	–	–	–	–	–	–	NI	NI	NI	+	–
CD14–a	B2	+	–	–	–	–	–	+	+	+	–	–	–	–	–	–	–	–	–	+	+	+	NI	NI	NI	–	+
JSL	D	+	–	–	–	–	–	+	+	+	+	–	–	–	–	+	–	–	–	–	–	–	–	+	–	–	+
E11	D	+	–	–	+	–	–	+	+	–	+	–	–	–	–	+	–	–	–	–	–	–	–	–	+	+	–
GM	D	–	+	–	–	–	–	+	+	+	+	–	–	–	–	–	–	–	–	–	+	–	+	+	–	+	–
PT1	B2	+	–	–	–	–	–	+	+	+	–	–	–	–	–	+	+	–	+	+	+	–	–	–	–	+	–
FBC	D	+	–	+	–	–	–	+	+	+	+	–	–	–	–	+	–	–	–	+	–	–	–	+	–	+	–
CPA	D	+	–	–	–	–	–	+	+	+	–	–	–	–	–	–	–	–	–	+	–	–	–	NI	NI	+	–
**AIEC TYPE**																											
HM605	B2	+	+	+	–	–	–	+	+	+	+	+	–	–	–	–	+	–	–	–	+	+	NI	NI	NI	NI	NI
NRG857c	B2	+	+	–	+	–	–	+	+	+	+	+	–	–	–	+	–	+	+	–	+	+	NI	NI	NI	NI	NI
**NON–CD STRAINS**																											
D1^*^	B1	+	–	–	–	–	–	+	–	–	–	+	–	–	–	–	–	–	–	–	–	–	–	–	–	–	–
D2^*^	B1	+	–	–	–	–	–	+	–	–	–	–	–	–	–	+	–	–	–	–	–	–	–	–	–	–	–
D3^*^	B2	–	–	–	–	–	–	+	+	+	–	–	–	–	–	–	–	–	–	+	+	–	–	–	–	–	–
D4^*^	B1	–	–	–	–	–	–	+	–	+	–	–	–	–	–	–	–	+	–	–	–	–	–	–	–	–	–
D5^*^	B2	+	–	–	+	–	–	+	+	+	–	–	–	–	–	–	–	–	–	+	+	–	–	–	–	–	–
D6^*^	D	+	+	+	+	+	–	+	+	+	–	–	–	–	–	–	+	–	–	+	–	–	–	–	–	–	–
D7^*^	A	+	–	–	–	–	–	+	–	+	–	–	–	–	–	–	+	+	–	+	–	–	–	–	–	–	–
D8^*^	A	+	–	–	–	+	–	+	–	+	–	+	–	–	–	–	+	–	–	+	–	–	–	–	–	–	–
D9^*^	A	+	–	–	–	–	–	+	–	+	–	+	–	–	–	–	+	–	–	+	–	–	–	–	–	–	–
D10^*^	D	+	+	+	–	–	–	+	+	+	–	–	–	–	–	–	+	–	–	+	–	–	–	–	–	–	–
D11^*^	A	+	–	–	+	–	–	+	–	+	–	–	+	–	–	–	–	–	–	+	+	–	–	–	–	–	–
C7–a^**^	A	+	–	+	–	–	–	+	–	–	–	–	–	–	–	–	–	–	–	+	–	–	–	–	–	–	–
14C05^**^	B1	+	–	+	+	–	–	+	–	–	–	+	–	–	–	–	–	–	–	–	–	–	–	–	–	–	–
15C02^**^	D	+	–	–	+	–	–	+	–	–	–	–	–	–	–	+	–	+	+	–	–	–	–	–	–	–	–
16C02^**^	A	+	+	–	–	+	–	+	+	+	+	–	–	–	–	+	+	+	+	–	+	–	–	–	–	–	–
19C01^**^	B2	+	+	–	+	–	–	+	+	+	–	+	–	–	–	+	+	–	–	+	+	–	–	–	–	–	–
22C05^**^	B2	+	+	–	–	+	–	+	+	–	–	–	+	–	–	–	+	–	–	–	–	–	–	–	–	–	–
23C01^**^	A	–	–	–	–	–	–	+	–	–	–	–	–	–	–	+	–	–	–	–	–	–	–	–	–	–	–
**COMMENSAL TYPE**																											
HS	A	+	–	–	–	–	–	+	–	–	–	–	–	–	–	–	–	–	–	–	–	–	–	–	–	–	–

*NI, without information*.

* *Non-CD strains from Chile*.

** *Non-CD strains from Spain*.

The dendrogram (Figure 1) revealed that even strain from the same geographic origin appeared intermixed as they did not group according to their origin. In addition, most *E. coli* clinical isolates-derived from patients with CD were associated with B2 and D genomic types (accounting for 84% of all strains, 27/32, *p* < 0.05). A very small percentage of CD strains were clustered within the genomic types B1 (3.1%, 1/32) and A (12.5%, 4/32). Furthermore, AIEC strains were also associated with the genomic groups B2 and D (92.3%, 12/13, *p* < 0.05), with the exception of AIEC strain CD9-a, which was associated with genomic group A. A significantly lower number of strains was isolated from group A in AIEC compared to non-AIEC strains (*p* < 0.05). On the other hand, isolates from non-CD patients were similarly distributed among these four main genomic types (38.8%, 7/18 in A; 22.2%, 4/18 in B1; 22.2%, 4/18 in B2; and 16.6%, 3/18 in D).

### Occurrence of virulence factors

Similarly, the distribution of virulence factors was diverse among the 32 CD strains studied. *fhuD* was present in all strains, including non-CD and reference strains, while *cdtB* was absent in all strains. *fimH* was present in almost all CD (96.8%, 31/32) and non-CD strains (83.3%, 15/18) studied. The most frequently detected factors were those coding for proteins associated with iron uptake, such as *fhuD* (32/32), *irp2* (25/32), and *chuA* (27/32). These factors were significantly more common in CD strains (69%) than in non-CD strains 33%) (OR: 4.4, 95% CI, *p* < 0.05; Table 4). Moreover, the most significant individual biomarker of CD strains was *chuA* (OR=8.5, 95% CI, *p* < 0.001). These results demonstrate that the presence of 3 iron transporters, and in particular chuA, is significantly associated with CD strains (Table 4). Furthermore, *chuA* is significantly associated with AIEC strains compared to non-AIEC strains (OR: 8.2, 95% CI, 0.95–69.74, *p* < 0.05). Nevertheless, in the case of adhesins, there was no observed association with CD. The adhesin gene *fimAv* was found to be negatively associated with CD (OR: 0.2, 95%CI: 0.04–0.96, *p* < 0.05). Toxin analysis showed that the most frequent toxin gene detected was *ratA*, which was present in 31.2% (10/32) of strains, and also allowed discrimination between CD strains (OR:7.7, 95%CI: 0.89–66.39, *p*-value > 0.05). The other toxin genes were rare in CD strains (Table 3), as were *cdtB* (0/32) and *cnfI* (1/32). The frequency of other virulence factors was as follows: *ibeA*: 9.3% (3/32), *gipA*: 12.5% (4/32), *pduC*; 50% (16/32), and *neuC*: 34% (11/32). *pduC* (propanediol catabolism) occurred in approximately 50% of the CD strains and was not significantly associated with CD (OR:2.6; 95%CI: 0.75–9.00, *p* > 0.1).

**Table 4:** *****Escherichia coli*** virulence genes in strains isolated from CD and non-CD and their association with Crohn's**.

**Virulence factors**	**Odds Ratio (OR)**	***P*-value**	**CD Association**
**ADHESINS**			
*fimH*	6.2 (0.59–64.72)	0.127	NS
*fimAv*	0.2 (0.04–0.96)	0.043^*^	Negative association
*aufA*	0.6 (0.15–2.33)	0.345	NS
*papC*	0.6 (0.15–2.33)	0.345	NS
*sfa/focDE*	–	–	–
*afaBC*	0.1 (0.0115–1.1043)	0.050	NS
**IRON TRANSPORTERS**			
*fhuD*	–	–	–
*chuA*	8.5 (2.21–32.56)	0.001^**^	Positive association
*irp2*	2.3 (0.64–8.05)	0.168	NS
three transporters *fhuD, chuA, irp2*	4.4 (1.28–15.09)	0.016^*^	Positive association
**TOXINS**			
*ratA*	7.7 (0.89–66.39)	0.034^*^	Positive association
*cvaC*	0.9 (0.23–3.19)	0.541	NS
*hlyA*	0.5 (0.06–4.15)	0.456	NS
*cnfl*	–	–	–
*cdtB*	–	–	–
**OTHER VIRULENCE FACTORS**			
*pduC*	2.6 (0.75–9.00)	0.108	NS
*neuC*	0.6 (0.20–2.13)	0.342	NS
*gipA*	0.5 (0.10–2.30)	0.303	NS
*ibeA*	0.8 (0.1–5.48)	0.599	NS
**SPATEs**			
CL-1	0.9 (0.28–2.89)	0.552	NS
CL-2	2.0 (0.58–7.02)	0.209	NS

Association between virulence factors and CD. Odds ratio measures how much the affected/non-affected rate increases with the factor. P-values, computed by Fisher Exact Probability Test (one-tailed), give the statistical significance of such effects. Last column indicates association S, Significant (significant (<0.05^*^); very significant (<0.01)^**^); NS, Non Significant for null (non-significant) association. Notation:– for not a number.

At least one class-1 or class-2 SPATE gene was found in 66% (21/32) of the CD strains and genes from both classes of SPATEs were present in 31% (10/32) of CD strains (Table 3). In AIEC strains this prevalence was 76% (10/13) and 38% (5/13), respectively. Class-1 SPATE genes were detected in 53% (17/32) of CD strains and class-2 SPATEs were detected in 44% (14/32) of CD strains. 62% (8/13) of AIEC strains contained class-1 SPATEs and 54% (7/13) contained class-2 SPATES. The presence of class-1 and class-2 SPATE genes was not significantly associated with CD or AIEC strains. On the other hand, 61%, (11/18) of non-CD strains harbored genes encoding at least one, class-1 or class-2 SPATEs, and 22% (4/18) more than one class of SPATE. The prevalence of SPATEs genes in non-CD strains was 55% (10/18) for class-1 and 28% (5/18) for class-2. With respect to the association between SPATE classes and the most frequent genomic types found in CD strains (B2 and D), no significant differences were detected in the presence of class-1 SPATEs in B2 (67%, 8/12) or D (53%, 8/15). However, significant differences were observed in the presence of class-2 SPATES between genomic types B2 (75%, 9/12) and D (33%, 5/15). The *neuC* and Class-2 SPATEs showed different results between Spanish and Chilean CD strains (*p* < 0.05) (Table 3).

### Genetic variations in *FimH*

**Figure 2:**
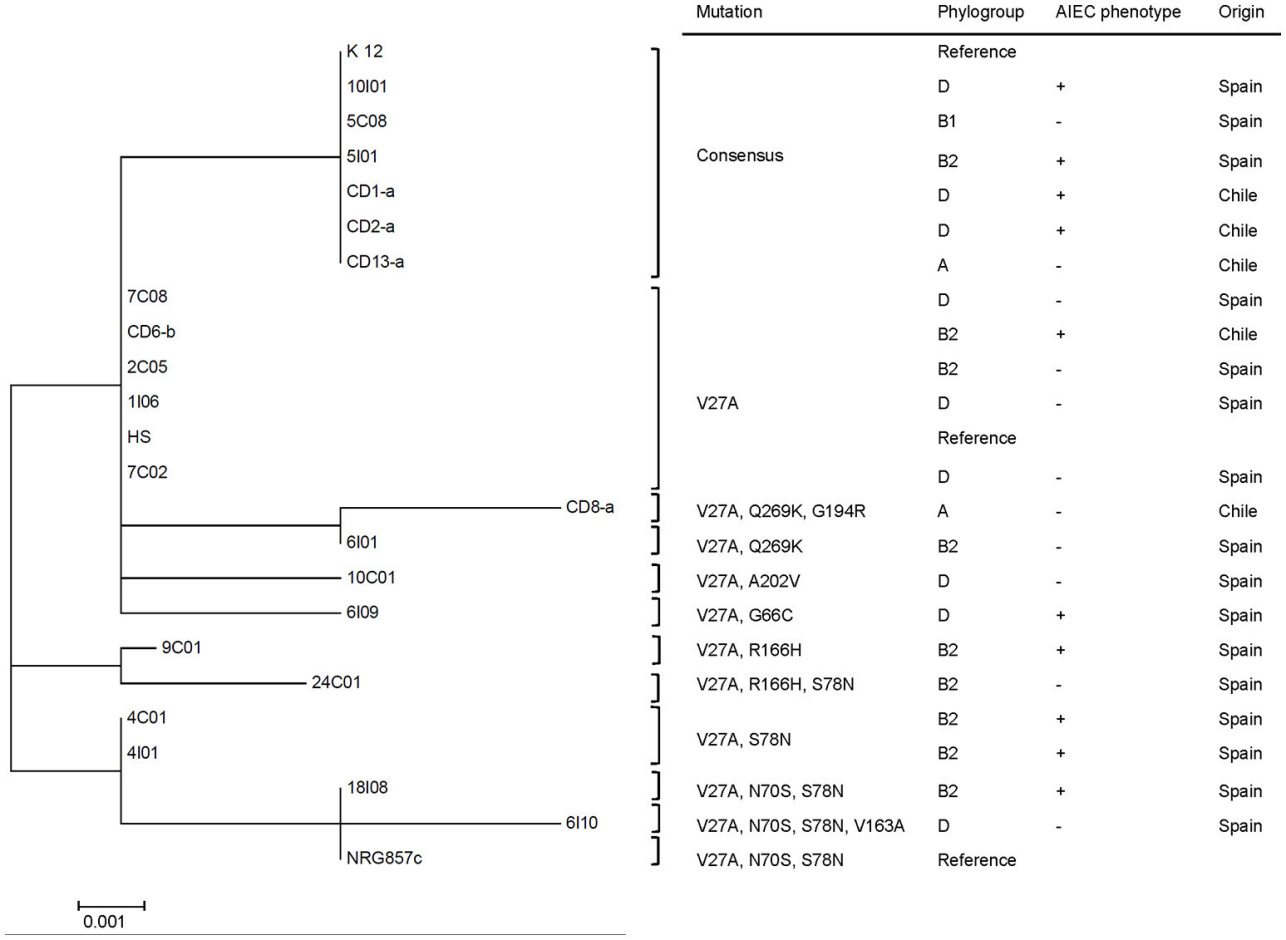
**A phylogenetic tree based on FimH amino acid sequences of ***E. coli*** strains isolated from patients with CD**. This tree was constructed using 21 complete FimH sequences from *E. coli* isolates, the AIEC reference strain NRG857c, *E. coli* commensal HS, and *E. coli* K-12 (FimH consensus sequence from reference strain K12), using the nearest-neighbor joining method. The phylogenetic tree presents three main clades and shows 10 different allelic variants.

Phylogenetic reconstruction of FimH, from 21 randomly selected CD *E. coli* isolates, indicated the presence of three major clades (Figure 2). Six out of 21 *E. coli* strains isolated from CD patients (5C08, 5I01, 10I01, CD1-a, CD2-a, and CD13-a) exhibited a FimH translated sequence identical to that of *E. coli* K12, whereas the remaining 15 showed the hot-spot mutation V27A. In this case, 9 amino-acid positions were affected by mutations (V27A, G66C, N70S, S78N, V163A, R166H, G194R, A202V, and Q269K) and generated 10 different allelic variants (Figure 2).

Altogether, the results indicate that the *E. coli* strains obtained from CD patients from Chile and Spain were genetically different in nature and did not group according to their geographic origin. In both, Chile and Spain groups of strains, the prevalence of genes associated with iron uptake was high. Some of these strains harbored FimH variants, which is in agreement with previous studies (Iebba et al., 2012). In addition, we found at least one SPATE class was found in 65.6% of *E. coli* strains isolated from CD patients.

## Discussion

*E. coli strains*, and in particular, the AIEC pathotype, have been identified by many authors as potentially implicated in the pathogenesis of CD (Darfeuille-Michaud et al., 1998; Boudeau et al., 1999; Glasser et al., 2001). The ability to adhere and invade epithelial cells, and survive intracellularly in macrophages characterizes this group of strains (Darfeuille-Michaud et al., 1998; Boudeau et al., 1999; Glasser et al., 2001; De la Fuente et al., 2014). Several virulence factors have been associated with these pathogenic properties in this pathotype (Conte et al., 2014; O'Brien et al., 2016) however they are not exclusive to AIEC strains (Martinez-Medina and Garcia-Gil, 2014). Interestingly, production of type 1 fimbriae appears to correlate with adhesiveness of AIEC strains and certain FimH variants confer them a higher adhesion to intestinal epithelial cells (Dreux et al., 2013). In addition, mucosa-associated *E. coli* strains isolated from CD patients were very heterogeneous and the most of them belonged to B2 and D genomic types, according to previous investigations (Kotlowski et al., 2007; Martinez-Medina et al., 2009). The *E. coli* strains isolated from CD were significantly distributed among phylogenetic groups D (*p* = 0.031), A (*p* = 0.037), and B1 (*p* = 0.050) (Table 3) when compared to non-CD strains. The phylogenetic group B2 was not significantly associated with any of the bacterial populations. The most common phylogenetic group among CD isolates was group D (47%) followed by groups B2 (37%), A (12.5%), and B1 (3.5%). Chilean and Spanish CD isolates were not significantly associated particularly with phylogenetic groups B2 (*p* = 0.058) or D (*p* = 0.369). In contrast, non-CD isolates were distributed among the following phylogenetic groups: A (36%), B1 (27%), and B2 and D, (18%). These results show significant differences among isolates of virulent strains associated with phylogenetic groups D and B2 from CD vs. non-CD (Table 3). These results are similar to those reported by Nowrouzian et al. (2006). It is possible that CD and non-CD subjects are transiently colonized by *E. coli* strains with different degrees virulence. However, it appears that those belonging the phylogenetic groups D and B2 are the most able of colonize and survive inside epithelial cells and macrophages in CD patients, particularly those harboring the *chuA* gene.

To date, all the features mentioned above have been studied in *E. coli* strains obtained from a particular geographic region. In this work however, we sought to investigate potential differences in mucosa-associated *E. coli* strains isolated from Chilean and Spanish patients with CD. To this end, we studied the genetic heterogeneity of these two *E. coli* populations, the presence of virulence factors known to be associated with CD *E. coli* and the occurrence of class-1 and class-2 SPATEs in these strains.

The clonal analyses of AIEC strains obtained from CD patients through PFGE showed a high degree of genetic variability, which is in agreement with the high variability of the distribution of virulence factors, and is similar to previous publications (Martinez-Medina et al., 2009; De la Fuente et al., 2014; O'Brien et al., 2016). The B2 and D genomic groups were highly prevalent among strains from CD subjects, as has been shown in previous reports (Kotlowski et al., 2007; Martinez-Medina et al., 2009; De la Fuente et al., 2014). Remarkably, we found that strains from different countries did not group according to their origin.

The most distinctive feature of the AIEC strains studied was that all of them had virulence genes related to iron uptake (*fhuD, chuA*, and *irp2*), which is in agreement with previous reports demonstrating AIEC strain enrichment with siderophores (Dogan et al., 2014). If a strain harbored the *fhuD, chuA*, and *irp2* transporters together, a high association with CD was noted (Table 4). The iron transporter gene *chuA* alone was also highly associated with CD (OR: 8.5, *p* = 0.001), as was the presence of the toxin gene *ratA* (OR: 7.7, *p* = 0.034).

The *fhuD* gene is common among pathogenic and non-pathogenic *E. coli* strains; however, *chuA* and *irp2* are less common among diarrheagenic and commensal *E. coli* strains. Nevertheless, both of these genes are present in ExPEC strains. The high prevalence of *fhuD, chuA*, and *irp2*, and its significance suggests that these three iron transporters or the presence of *chuA* alone may act as predictors of CD strains and AIEC isolated patients with CD. They may be relevant biological or diagnostic markers, which could be used to characterize and identify invasive strains isolated from CD patients. Another prevalent gene in the strains studied was *pduC*, which encodes proteins required for propanediol catabolism, was present in 50% of CD strains and in 66% of AIEC strains. *pduC* has been also described previously in the *S. enterica* serovar Thyphimurium (Bobik et al., 1997). The prevalence of *pduC* in AIEC strains in this study was similar to that reported by Dogan et al. who suggested that its presence in AIEC strains seems to be related to their persistence in macrophage cells (Dogan et al., 2014). However, *pduC* was not significantly associated with CD strains. The *neuC* gene is necessary for sialic acid synthesis, which is a carbohydrate monomer component of the K1 capsule, and is a virulence factor that is essential in *E. coli* strains causing meningitis in newborns (Vann et al., 2004). In addition, the K capsule contributes to intracellular replication of uropathogenic *E. coli* (UPEC) strains (Goller and Seed, 2010). *neuC* was present in 31% of CD strains and it is possible that NeuC plays a similar role in AIEC strains, contributing to its intracellular survival.

In agreement with previous studies, the gene encoding the FimH adhesin of type 1 fimbriae was present in almost all *E. coli* strains (De la Fuente et al., 2014). In AIEC strains this adhesin is considered a virulence factor and is responsible for mannose-dependent bacterial adherence and invasion of epithelial cells (Barnich et al., 2007; Brument et al., 2013; Dreux et al., 2013). FimH mediates the adhesion of AIEC strains to the apical surface of the ileal epithelium, specifically to the CEACAM6 protein, which is overexpressed in patients with CD (Barnich et al., 2007; Carvalho et al., 2009; Barnich and Darfeuille-Michaud, 2010). Different *fimH* allelic types occur in AIEC strains, which may increase adhesion to T84 cells and confer colonization advantages in the intestinal tract (Iebba et al., 2012; Dreux et al., 2013). Moreover, allelic replacement of the *fimH* gene in the AIEC LF82 genetic background by *fimH* from non-pathogenic K12 strains, significantly reduced bacterial colonization and colitis signs in CEABAC10 transgenic mice expressing human CEACAM6 (Dreux et al., 2013). Some of the mutations reported here, such as V27A, N70S, and S78N, have been previously reported in UPEC strains. These polymorphisms are considered to be potential patho-adaptations that may confer significant advantages in regards to bacterial epithelial colonization (Eris et al., 2016). Such patho-adaptative polymorphisms were also observed in *E. coli* strains obtained from pediatric patients with inflammatory bowel disease (IBD) (Iebba et al., 2012). V27A and G66C mutations in FimH were associated with CD, and G66S, N70S, and S78N were more prevalent in the B2 and D genomic groups (Iebba et al., 2012). However, Dreux et al. suggested that the V27A polymorphism was not associated with the AIEC pathotype (Dreux et al., 2013). The V27A mutation found in strains isolated from patients with IBD and CD was present in the isolates analyzed here. In our study, we confirmed the presence of several previously described mutations in AIEC strains (V27A, G66C, N70S, S78N, V163A, R166H, G194R, A202V, and Q269K) (Iebba et al., 2012; Dreux et al., 2013). However, the prevalence of V27A in the FimH gene in mucosa-associated *E. coli* isolated from Chilean and Spanish patients was 71%. Furthermore, the highly invasive AIEC strains, CD2-a and CD1-a (De la Fuente et al., 2014), lack any of the previously reported patho-adaptative polymorphisms. It is likely that adhesion and invasion mechanisms associated with these strains are mediated by other adhesins. Our results are consistent with previous report by Desilets et al. (2016), who suggested that there is no strict association between the previously described polymorphisms of FimH in the AIEC pathotype and its superior adhesion and invasion ability (Desilets et al., 2016).

The prevalence of SPATE genes in mucosa-associated *E. coli* isolates was also analyzed. SPATE function is varied, they can have intracellular or extracellular protein substrates, and are involved in numerous biological functions, such as those implicated in cytoskeleton stability, autophagy or innate and adaptive immunity (Ruiz-Perez and Nataro, 2014). These proteins have evolved distinct functions that are adaptive to the particular niche occupied by the pathogen (Henderson and Nataro, 2001). We found that 65% of the CD and 73% of AIEC strain isolates presented at least one SPATE gene class. This prevalence is high, but not significantly higher than in the control group (61.1%). These results are similar to those reported by Souza et al., who did not find a significant difference in the presence of SPATEs between CD and control samples (De Souza et al., 2012).

Class-1 SPATE genes were present in 53.1% of CD strains. To date, there are no reports that associate the presence of class-1 SPATEs with CD. Yet, the cytotoxic activity and cellular damage associated with these proteins may promote the intestinal inflammation characteristic of CD. In this context, cytotoxic SPATEs have been found almost exclusively in microbial pathogens (*Shigella*, enteroagregative *E. coli* and enteroinvasive *E. coli*) that induce mucosal damage and inflammation (Boisen et al., 2009). Class-2 SPATE genes were present in 43% of CD isolates and 61.5% of AIEC strains. For most of the proteins of this class, little is known about their possible role in pathogenesis. However, most of the class-2 SPATEs studied to date display proteolytic activity on mucins (Ruiz-Perez and Nataro, 2014), which facilitates the interactions of the bacteria with the intestinal mucosa. Recently, these proteins have been reported to harbor lectin-like properties, conferring the capability of agglutination and adhesion to diverse white cells, as well as the ability to modulate the immune response at distinct levels, cleaving chemokines, complement proteins, adhesion proteins, and co-stimulatory molecules, all involved in malignancy and inflammatory processes (Ayala-Lujan et al., 2014), which may contribute to AIEC pathogenicity.

In conclusion, *E. coli* strains isolated from CD patients from Spain and Chile show a high degree of genetic variability. They also show significant variability in the virulence genes present, with the exception of genes involved in iron uptake and the *ratA* toxin gene, which were highly represented in CD strains and are likely to play a relevant role in AIEC pathogenesis. Furthermore, we did not observe an association between the FimH polymorphism and the adhesion and invasion ability of the strains studied. On the other hand, class-1 and -2 SPATE genes were not significantly more prevalent in CD strains or non-CD strains. This study highlights the potential of virulence genes present in CD strains to play a significant role in the pathogenesis of this disease. It is important to highlight that of all of the *E. coli* strains isolated from patients with CD, both in Chile and Spain, 40% (13/32) were identified as AIEC strains, whose classical characteristics are adhesion to and invasion of cells in culture and resistance to phagocytosis by macrophages.

## Author contributions

SC and WS, Study design, experimental analysis, and manuscript writing. FDC, Bioinformatics analysis. MDF, Isolation of Chilean AIEC strains from biopsies. RQ, Collection of biopsies from Chilean patients. MH, Study design and manuscript writing. RM, Isolation of Spanish AIEC strains from biopsies. DG and SK, Collection of biopsies from Spanish patients. JG, Data analysis and English edition. RA, Statistic analysis. RR, Bioinformatics analysis and protocol planning in Spain. RV, Study design and supervision, team coordination among Chile, and Spain, analysis of results, manuscript writing and edition.

### Conflict of interest statement

The authors declare that the research was conducted in the absence of any commercial or financial relationships that could be construed as a potential conflict of interest.
